# P-1471. Impact of PCV10 Introduction on Nasopharyngeal Carriage of Streptococcus pneumoniae in Healthy Children in Rural and Urban Nepal

**DOI:** 10.1093/ofid/ofaf695.1657

**Published:** 2026-01-11

**Authors:** Madhav Chandra Gautam, Sonu Shrestha, Sanjeev Man Bijukchhe, Meeru Gurung, Bhishma Pokhrel, Sarah Kelly, David R Murdoch, Maria Deloria Knoll, Dominic F Kelly, Shrijana Shrestha, Andrew J Pollard

**Affiliations:** Patan Academy of Health Sciences, Kathmandu, Bagmati, Nepal; University of Oxford, Oxford, England, United Kingdom; Patan Academy of Health Sciences, Kathmandu, Bagmati, Nepal; Patan Academy of Health Sciences, Kathmandu, Bagmati, Nepal; Patan Academy of Health Sciences, Kathmandu, Bagmati, Nepal; University of Oxford, Oxford, England, United Kingdom; University of Otago, Christchurch, Christchurch, Canterbury, New Zealand; Johns Hopkins Bloomberg School of Public Health, Baltimore, Maryland; University of Oxford, Oxford, England, United Kingdom; Patan Academy of Health Sciences, Kathmandu, Bagmati, Nepal; University of Oxford, Oxford, England, United Kingdom

## Abstract

**Background:**

The ten-valent pneumococcal conjugate vaccine (PCV10) was introduced in Nepal in 2015. We compared the nasopharyngeal carriage of PCV10and non-PCV10 serotypesof pneumococcusbetween pre-vaccine (2014-2015) and post-vaccine (2016-2019) years in two different regions of Nepal.

**Methods:**

Nasopharyngeal samples obtained in healthy Nepalese children aged 6-59 months in urban (Patan, Kathmandu) and 6-23 months in rural (Okhaldhunga) settings were transported in STGG (Skim Milk-Tryptone-Glucose-Glycerol) media, cultured for pneumococcus and serotyped by the Quellung method.

**Results:**

PCV10-type prevalence decreased from 27.67% in rural and 19.08% in urban children pre-vaccine to 9.73% and 9.18% post-vaccine, respectively. Pre-vaccine, the most frequently found serotypes in both settings were 19F, 6B, 14. Post-vaccine, the non-PCV10 serotypes were more common. Significant increases were observed in additional PCV13 serotypes 3, and 19A, in both settings. In addition, in the urban setting, non-vaccine serotypes 15C, 34, 13, 23B and 33B increased, whereas, serotypes 10A, 23A and 33F increased in the rural setting. The carriage prevalence has decreased for most of the PCV10-type serotypes in both the settings except 7F, 9V, 4 and 5, which did not show significant decrease.

**Conclusion:**

Since the introduction of PCV10, carriage prevalence of most of the PCV10 serotypes have reduced and non-PCV10 serotypes have increased in both settings. However, due to increase in non-PCV10 serotypes, replacement disease may occur in the future. Thus continued monitoring of changes in the nasopharyngeal carriage of vaccine type pneumococcal serotypes is important when assessing vaccine impact.

**Disclosures:**

All Authors: No reported disclosures

